# Ionizing radiation, ion transports, and radioresistance of cancer cells

**DOI:** 10.3389/fphys.2013.00212

**Published:** 2013-08-14

**Authors:** Stephan M. Huber, Lena Butz, Benjamin Stegen, Dominik Klumpp, Norbert Braun, Peter Ruth, Franziska Eckert

**Affiliations:** ^1^Department of Radiation Oncology, University of TübingenTübingen, Germany; ^2^Department of Pharmacology, Toxicology and Clinical Pharmacy, Institute of Pharmacy, University of TübingenTübingen, Germany

**Keywords:** radiation therapy, cell cycle, DNA repair, ion channels

## Abstract

The standard treatment of many tumor entities comprises fractionated radiation therapy which applies ionizing radiation to the tumor-bearing target volume. Ionizing radiation causes double-strand breaks in the DNA backbone that result in cell death if the number of DNA double-strand breaks exceeds the DNA repair capacity of the tumor cell. Ionizing radiation reportedly does not only act on the DNA in the nucleus but also on the plasma membrane. In particular, ionizing radiation-induced modifications of ion channels and transporters have been reported. Importantly, these altered transports seem to contribute to the survival of the irradiated tumor cells. The present review article summarizes our current knowledge on the underlying mechanisms and introduces strategies to radiosensitize tumor cells by targeting plasma membrane ion transports.

## Introduction

Increasing pieces of evidence strongly indicate that ion transports across biological membranes fulfill functions beyond those described by classical physiology such as epithelial transports and neuronal or muscle excitability. More and more, it turns out that ion transports are involved in virtually all cell-biological processes. By modifying the chemistry, electricity and mechanics of cells, ion transports directly interact with cellular biochemistry and constitute signaling modules that are capable of altering protein function, gene expression (Tolon et al., 1996) and epigenetics (Lobikin et al., 2012). Moreover, ion transport-generating proteins such as ion channels have been identified to directly signal in macromolecular complexes with, e.g., surface receptors and downstream kinases (Arcangeli, 2011), or to directly bind to DNA as transcription factors (Gomez-Ospina et al., 2006).

Over the past two decades, ion transports came more and more in the focus of oncological research. Increasingly, data accumulate indicating tumor-suppressing as well as oncogenic functions of ion transport processes. In particular, ion transports have been identified as key regulators of neoplastic transformation, malignant progression, tissue invasion and metastasis (for review see Huber, 2013). Most recent data suggest that ion transports may also contribute to therapy resistance especially to radioresistance of tumor cells. The second chapter of this review article aims at giving an overview of those data. Since worldwide, only a handful of laboratories including ours are working in this research field only few data on ion transports in radioresistance are available and in most cases, the underlying molecular mechanisms of the observed phenomena remain ill-defined. Because tumor hypoxia is a major obstacle in radiotherapy, the second chapter also includes ion transports in the mitochondria that confer hypoxia resistance to normal tissue and probably also to tumor cells. At the end, this article provides some ideas how the acquired knowledge might be harnessed in the future for new strategies of anti-cancer therapy that combine ion transport-targeting and radiotherapy. To begin with, a brief introduction into radiotherapy and its radiobiological principles is given in the next paragraphs.

## Radiotherapy

According to the German Cancer Aid, 490,000 people in Germany are diagnosed with cancer every year (German-Cancer-Aid, 2013a) (data originating from February 2012), 218,000 die from their disease. About half of all cancer patients receive radiation treatment, half of all cures from cancer include radiotherapy (German-Cancer-Aid, 2013b). Radiotherapy is one of the main pillars of cancer treatment together with surgery and systemic therapy, mainly chemotherapy. Examples for curative radiotherapy without surgery are prostate (Eckert et al., 2013; Kotecha et al., 2013) and head and neck cancer (Glenny et al., 2010). Preoperative radiotherapy is applied in rectal cancer (Sauer et al., 2012), postoperative treatment in breast cancer (Darby et al.). Yet, also rare tumor entities like sarcoma and small cell carcinoma are treated with radiotherapy (Eckert et al., 2010a,b; Muller et al., 2012). Despite modern radiation techniques and advanced multimodal treatments local failures and distant metastases often limit the prognosis, especially due to limited salvage treatments (Muller et al., 2011; Zhao et al., 2012).

### Intrinsic and hypoxic radioresistance

Radiation therapy impairs the clonogenic survival of tumor cells mainly by causing double strand breaks in the DNA backbone. The number of double strand breaks increases linearly with the absorbed radiation dose (unit Gray, Gy). The intrinsic capacity to repair these DNA damages by non-homologous end joining or homologous recombination determines how radio resistant a given tumor cell is. Irradiated tumor cells which leave residual DNA double strand breaks unrepaired lose their clonogenicity meaning that these cells cannot restore tumor mass. Ion transports may directly be involved in the cellular stress response to DNA damage by controlling cell cycle, metabolic adaptations or DNA repair and, thus, contribute to intrinsic radioresistance and the survival of the tumor cell.

Besides intrinsic factors, the microenvironment influences the radiosensitivity of a tumor. Hypoxic areas frequently occur in solid tumors. Hypoxic tumor cells, however, are somehow “protected” from radiotherapy [reviewed in Harada (2011)]. This is because ionizing radiation generates directly or indirectly radicals in the deoxyribose moiety of the DNA backbone. In a hypoxic atmosphere, thiols can react with those DNA radicals by hydrogen atom donation which results in chemical DNA repair. In the presence of oxygen, in contrast, oxygen fixes radicals of the deoxyribose moiety to strand break precursors (Cullis et al., 1987). This so called oxygen effect radiosensitizes tumor cells by a factor of two to three (oxygen enhancement ratio) as compared to the hypoxic situation (Langenbacher et al., 2013). Accordingly, patients with hypoxic tumors who undergo radiotherapy have a worse prognosis than those with normoxic tumors [e.g., cervical cancer (Fyles et al., 2002, 2006)]. Notably, ion transport processes have been identified as important players in the adaptation of tumor cells to a hypoxic microenvironment. Hence, ion transports via adaptation to hypoxia also indirectly contribute to the radioresistance of tumors.

In radiotherapy, fractionated treatment regimens have been established which may reoxygenate and thereby radiosensitize the irradiated tumor during therapy time. In addition, fractionated radiotherapy spaces out the single fractions in a way that allows DNA repair of normal tissue, that re-distribute cell cycle of the tumor cells in more sensitive phases and that minimize repopulation of the tumor during therapy. The next paragraphs will give an introduction to the underlying radiobiology.

### Fractionated radiation therapy. repair, reoxygenation, redistribution, and repopulation

Early in historic development of radiotherapy fractionation was introduced as a means to limit side effects when giving therapeutic radiation doses (Bernier et al., 2004). Standard fractionation is defined as single doses of 1.8–2 Gy, once daily, 5 days per week.

The principal rationale for fractionation is based on the fact that recovery after radiation is better in normal tissue than in tumors, especially concerning late reacting tissues responsible for late side effects of radiotherapy (Jones et al., 2006) such as fibrosis, damage of spinal cord and brain, as well as most inner organs. Radiation with high single doses is only possible without increased side effects if the radiation field can be confined to the tumor (e.g., stereotactic radiotherapy of brain metastases [Rodrigues et al., 2013) and SBRT, stereotactic body radiation therapy (Grills et al., 2012)]. Yet, many situations in radiation oncology such as adjuvant treatment or irradiation of nodal regions require irradiation of significant volumes of normal tissue.

#### Alpha-beta ratios

Acute effects of ionizing irradiation on clonogenic cell survival *in vitro* as well as on late toxicity of the normal tissue in patients which underwent radiotherapy are described by the linear-quadratic model (Barendsen, 1982; Dale, 1985). The mathematical fit of the clonogenic survival (late toxicity) is calculated as follows: *N* = *N*_0_ × *E*^−(α*D*−β*D*^∧^2)^ with *N* being the number of surviving cells (patients without late toxicity), N_0_ being the initial number of cells (number of patients receiving radiotherapy), α [1/Gy] and β [1/Gy^∧^2] being cell (tissue)-specific constants and D the delivered radiation dose. Low alpha-beta ratios (α/β) [Gy] as determined for many normal tissues indicate that dose fractionation in daily fractions of usually 2 Gy increases survival and decreases late toxicity as compared to a single equivalent dose. Tumors with high alpha-beta ratios, in contrast do not benefit from fractionation. For some tumors such as squamous cell carcinoma of the head and neck there is even a rationale for hyperfractionated radiotherapy with twice daily irradiation of 1.2–1.4 Gy per fraction [reviewed in Nguyen and Ang (2002)]. The theoretical advantage has been confirmed in clinical trials [e.g., EORTC trial 22791 in advanced head and neck cancer Horiot et al. (1992)]. Different fractionation schedules for distinct clinical situations are applied for example in whole-brain radiotherapy. In prophylactic radiation 2–2.5 Gy fractions are applied to limit neurocognitive deficits (Auperin et al., 1999; Le Pechoux et al., 2011; Eckert et al., 2012). For therapeutic radiation 3 Gy fractions or even 4 Gy fractions are preferred in a palliative setting and limited life expectancy to shorten the treatment time to 5 or 10 days (Lutz, 2007; Rades et al., 2007a,b).

#### Reoxygenation

As mentioned above, fractionated radiation may also lead to reoxygenation of the tumor during therapy (Withers, 1975; Pajonk et al., 2010). Blood vessels of tumors lack normal architecture and are prone to collapse whenever tissue pressure of the expanding tumor mass increases. This aggravates tumor mal-perfusion and accelerates intermittent or chronic tumor hypoxia. Being sublethal as related to the whole tumor, single radiation fractions in the range of 2 Gy kill a significant percentage of the tumor cells which give rise to tumor shrinkage. Shrinkage, in turn, is thought to increase blood and oxygen supply of the tumor by improving vessel perfusion and by increasing the ratio of vascularization and the residual tumor mass (Maftei et al., 2011; Narita et al., 2012). Increased oxygenation then reverses hypoxic radioresistance of the tumor and improves the therapeutic outcome of radiotherapy.

#### Redistribution and repopulation

The sensitivity to radiotherapy during cell cycle differs, being highest in M and lowest in late S phase of cell cycle (Pawlik and Keyomarsi, 2004). Often depending on p53 function, irradiated tumor cells accumulate in G_1_ or G_2_ phase of cell cycle to repair their DNA damages. In a radiation dose-dependent manner, irradiated cells are released from cell cycle arrest and re-enter cell cycling and tumor repopulation. Importantly, repopulation after irradiation is often accelerated probably due to selection of more aggressive tumor cells (Marks and Dewhirst, 1991). Fractionated radiation regimes aim to re-distribute tumor cells in a more vulnerable phase of the cell cycle in the time intervals between two fractions and to impair repopulation (Pawlik and Keyomarsi, 2004).

### Cancer stem cells (CSCs)

Cancer stem cells (CSCs) may resist radiation therapy [for review see Pajonk et al. (2010)]. Mechanisms that might contribute to the relative resistance of CSCs as compared to the non-CSC cells of a given tumor include (i) higher oxidative defense and, therefore, lower radiation-induced insults, (ii) activated DNA checkpoints resulting in faster DNA repair, and (iii) an attenuated radiation-induced cell cycle redistribution. Fractionation regimes are designed that way that the macroscopically visible bulk of tumor cells (i.e., the non CSCs) and not the rare CSCs become redistributed into a more vulnerable phase of cell cycle between two consecutive fractions of radiotherapy. Finally, radiation therapy is thought to switch CSCs from an asymmetrical into a symmetrical mode of cell division; i.e., a CSC which normally divides into a daughter CSC and a lineage-committed progenitor cell is induced by the radiotherapy to divide symmetrically into two proliferative stem daughter cells. This is thought to accelerate repopulation of the tumor after end of radiotherapy (Pajonk et al., 2010).

In summary, fractionated radiotherapy may radio sensitize tumor cells by reoxygenation of the tumor and redistribution of the tumor cells in more vulnerable phases of cell cycle while protecting at the same time normal tissue if the alpha-beta ratio of the tumor exceeds that of the normal tissue. On the other hand, the applied fractionation protocols might spare CSCs due to their radiobiology that differs from that of the bulk of non-CSCs. Furthermore, single radiation fractions apply sublethal doses of ionizing radiation. Data from *in vitro* and animal studies suggest that sublethal doses of ionizing radiation may stimulate migration and tissue invasion of the tumor cells. Translated into the *in vivo* situation, this might imply that cells at the edge of solid tumors might be stimulated by the first radiation fractions to migrate out of the target volume of radiation resulting in survival of the evaded cells and tumor relapse. Moreover, if radiation fractions further induce tumor cell invasion into blood or lymph vessels, fractionated radiotherapy regimes might also boost metastases. As described in the next paragraphs, ion transports fulfill pivotal functions in cell migration especially in radiation-induced migration.

## Ion transports and radioresistance

Ion transports can be assessed by tracer-flux measurements, fluorescence microscopy/photometry using ion species-specific fluorescence dyes such as the Ca^2+^-specific fluorochrome fura-2, as well as by electrophysiological means. The latter can be applied if ion transports are electrogenic. Measurements of ion transports during treatment with ionizing radiation are hardly feasible. Reported electrophysiological *in vitro* data on irradiated tumor cells indicate that radiation-induced transport modifications may occur instantaneously and may last up to 24 h post irradiation (Kuo et al., 1993). They further suggest that these modifications may be induced by doses used for single fractions in the clinic (Steinle et al., 2011). The following paragraphs summarize radiation-induced transport modifications as observed in *in vitro* studies on tumor cell lines and their putative contribution to the radioresistance of tumor cells. Whether these processes may indeed underlie therapy failure in tumor patients can only be answered if more data from tumor mouse models and clinical trials become available.

Tumor cells have been proposed to adapt either a “Grow” or a “Go” phenotype in dependence on changes in their microenvironment. When developing a certain mass, growing solid tumors are prone to become malperfused because of the insufficient tumor vasculature. As a consequence of malperfusion, microenvironmental stress by hypoxia, interstitial nutrient depletion, and low pH increases (Stock and Schwab, 2009; Hatzikirou et al., 2012) which is thought to trigger at a certain point the induction of the “Go” phenotype. By migration and tissue invasion “Go” tumor cells may evade the locally reined stress burden and resettle in distant and less hostile regions. Once re-settled, tumor cells may readapt the “Grow” phenotype by reentering cell cycling and may establish tumor satellites in more or less close vicinity of the primary focus. Moreover, this stress evasion may lead to metastases if the “Go” cells invade into blood or lymph vessels.

Migration and tissue invasion are directed by extracellular hapto- and chemotactic signals which trigger preset “Go” programs (Schwab et al., 2007, 2012). The latter comprise intracellular signaling, cellular motor functions including cell volume changes and cytoskeletal dynamics, as well as extracellular matrix digestion and reorganization. Ion transports have been suggested to contribute to all of these processes (Schwab et al., 2007, 2012). As a matter of fact, highly invasive and metastatic phenotypes of tumor cells often show aberrant activity of certain ion transports. The following paragraphs describe the role of these ion transports in particular of those across the plasma membrane using the example of glioblastoma cells.

### Motor function

Glioblastoma cells exhibit a highly migrative phenotype and “travel” long distances throughout the brain (Johnson et al., 2009). Primary foci of glioblastoma show, therefore, even at early stages of diagnosis a characteristic diffuse and net-like brain infiltration (Niyazi et al., 2011). Tumor margins are often not definable and complete surgical tumor resection as well as capture of all residual tumor cells by the radiation target volume is hardly possible (Weber et al., 2009). This results in therapy failure accompanied by very bad prognosis for the survival of the patient in almost all cases of glioblastoma (Niyazi et al., 2011). Glioblastoma cells typically migrate into the surrounding brain parenchyma primarily by using nerve bundles and the vasculature as tracks. The close vicinity to the vasculature has the advantage for the migrating glioblastoma cell of a continuous and sufficient supply of oxygen, nutrients, growth factors, chemokines, and cytokines (Montana and Sontheimer, 2011). Glioblastoma cells have to squeeze through very narrow interstitial spaces during their brain invasion along those tracks. This requires highly effective local cell volume decrease and re-increase procedures. Notably, glioblastoma cells are capable to lose all unbound cell water leading to maximal cell shrinkage (Watkins and Sontheimer, 2011). Unusually high cytosolic Cl^−^ concentrations (100 mM) provide the electrochemical driving force for this tremendous cell volume decrease. The cytosolic Cl^−^ concentration is built up highly above its electrochemical equilibrium concentration by the Na/K/2Cl cotransporter NKCC1 (Haas and Sontheimer, 2010; Haas et al., 2011) allowing glioblastoma cells to utilize Cl^−^ as an osmolyte.

Local regulatory volume increase and decrease have been proposed to drive migration mechanics. The latter is generated by the loss of Cl^−^ and K^+^ ions along their electrochemical gradients followed by osmotically obliged water fluxes. Involved transporters probably are ClC-3 Cl^−^ channels (Olsen et al., 2003; Cuddapah and Sontheimer, 2010; Lui et al., 2010), Ca^2+^-activated high conductance BK (Ransom and Sontheimer, 2001; Ransom et al., 2002; Sontheimer, 2008) as well as intermediate conductance IK K^+^ channels (Catacuzzeno et al., 2010; Sciaccaluga et al., 2010; Ruggieri et al., 2012) and AQP-1 water channels (Mccoy and Sontheimer, 2007; Mccoy et al., 2010). To a lower extent, K^+^ and Cl^−^ efflux is probably also mediated by KCC1-generated cotransport (Ernest et al., 2005). These transports are crucial for glioblastoma migration since either transport blockade inhibits glioblastoma cell migration and invasion (Ernest et al., 2005; Mcferrin and Sontheimer, 2006; Catacuzzeno et al., 2010; Haas and Sontheimer, 2010; Lui et al., 2010; Sciaccaluga et al., 2010).

Notably, Ca^2+^-activated BK (Ransom and Sontheimer, 2001; Liu et al., 2002; Ransom et al., 2002; Weaver et al., 2006) and IK K^+^ channels (Ruggieri et al., 2012) are ontogenetically down-regulated or absent in mature glial cells but up-regulated with neoplastic transformation and malignant tumor progression as shown in expression studies in human glioma tissue. Moreover, glioblastoma cells up-regulate a unique splice variant of the BK channel (Liu et al., 2002) which exhibits a higher Ca^2+^ sensitivity than the other isoforms (Ransom et al., 2002) and is indispensable for glioblastoma proliferation *in vitro*. Similarly, ClC-3 Cl^−^ channels are mal-expressed in glioblastoma tissue where they traffic, in contrast to normal tissue, to the plasma membrane (Olsen et al., 2003). The predominant (surface) expression of ClC-3 and the BK splice variant by glioblastoma cells renders both channel types putative glioblastoma-specific therapeutic targets.

### Evasion from radiation stress

**Figure 1:**
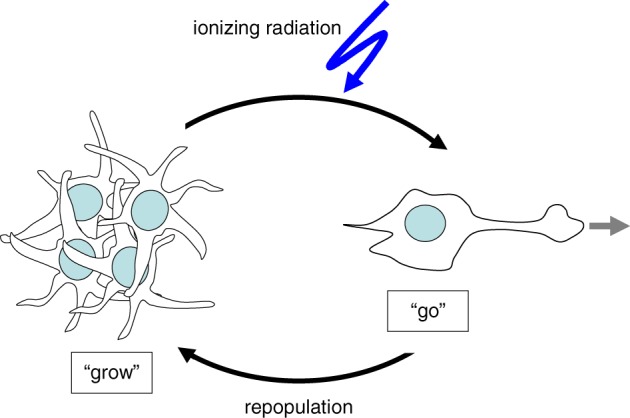
**Cellular stress such as ionizing radiation induces the switch from “Grow” to “Go” phenotype**.

External beam radiation may induce the “Go” phenotype in tumor cells similarly to the situation described for stress arising from an adverse tumor microenvironment (Figure 1). Ionizing radiation at doses used in single fractions during fractionated radiotherapy has been demonstrated *in vitro* and by a mouse study (Wild-Bode et al., 2001) to induce migration, invasion and spreading of head and neck squamous carcinoma (Pickhard et al., 2011), lung adenocarinoma (Jung et al., 2007; Zhou et al., 2011), meningioma (Kargiotis et al., 2008), medulloblastoma (Asuthkar et al., 2011), and glioblastoma cells (Wild-Bode et al., 2001; Wick et al., 2002; Badiga et al., 2011; Canazza et al., 2011; Rieken et al., 2011; Steinle et al., 2011; Kil et al., 2012; Vanan et al., 2012). The phenomenon of radiation-stimulated migration might be particularly relevant for highly migrating and brain-infiltrating glioblastoma cells.

After macroscopic complete resection glioblastoma is usually treated by adjuvant radiotherapy of the tumor bed applying 54–60 Gy in daily fractions of 1.8–2 Gy combined with temozolomide (Stupp et al., 2005). The median progression-free survival after therapy ranges between 5 and 7 months (Stupp et al., 2005). The recurrence of glioblastoma is typically observed within the former target volume of the adjuvant fractionated radiotherapy. This might be due either to a high intrinsic radioresistance of the glioblastoma cells or to re-invasion of tumor cells into the area of the irradiated primary. One might speculate that this necrotic area, meanwhile cleared by phagocytes, offers optimal growth conditions for such re-invading tumor cells. In this latter scenario, re-invading cells might be recruited from glioblastoma (stem) cells pre-spreaded prior to radiotherapy onset in areas outside the target volume, or from cells that successfully evaded during radiation therapy.

**Figure 2:**
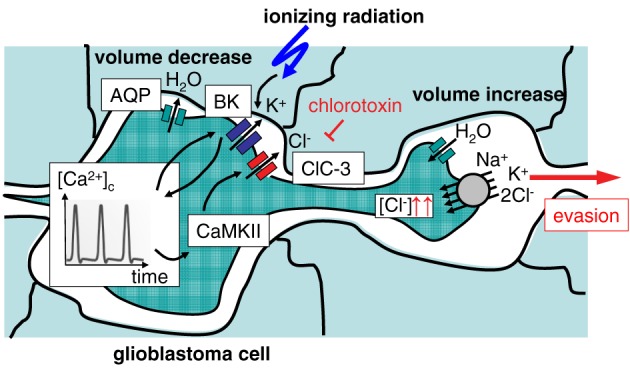
**Ionizing radiation (IR)-induced activation of BK K^+^ channels results in evasion of glioblastoma cells from radiation stress**. Activation of BK channels occurs upstream of CaMKII and ClC-3 Cl^−^ channels. The Na^+^/K^+^/2Cl^−^ cotransporter accumulates Cl^−^ above its electrochemical equilibrium. Cl^−^ is used as osmolyte for volume decrease.

Radiation-induced up-regulation of integrin- (Wild-Bode et al., 2001; Nalla et al., 2010; Canazza et al., 2011; Rieken et al., 2011), VEGF- (Sofia Vala et al., 2010; Badiga et al., 2011; Kil et al., 2012), EGF- (Kargiotis et al., 2008; Pickhard et al., 2011) or/and TGFbeta signaling (Canazza et al., 2011; Zhou et al., 2011) has been proposed to promote tumor cell migration. Downstream ion transport processes have been reported for glioblastoma cells (Steinle et al., 2011). In this study, BK K^+^ channel activation and subsequent BK-dependent activation of the CaMKII kinase were identified as key triggers of radiation-induced migration (Steinle et al., 2011). Additionally, ClC-3 anion channels were identified as downstream targets of radiation-induced CaMKII activity (Huber, 2013). This suggests on the one hand motor function (i.e., volume decrease) of radiation-induced BK and ClC-3 currents, on the other hand, it points to a signaling function of BK channels in the programming of radiation-stimulated glioblastoma migration (Figure 2). Similar to the situation in migrating glioblastoma cells, radiation-induced plasma membrane K^+^ currents and downstream CaMKII activation have been defined as key signaling events in cell cycle control of irradiated leukemia cells as introduced in the following paragraphs.

### DNA repair

Survival of irradiated tumor cells critically depends on DNA repair. This involves cell cycle arrest, elevated energy consumption, chromatin relaxation, and formation of repair complexes at the site of DNA damage. Recent *in vitro* observations suggest that radiation-induced ion transports may contribute to these processes in an indirect manner.

#### Cell cycle control

Survival of irradiated human leukemia cells depends on Ca^2+^ signaling. Radiation reportedly stimulates Ca^2+^ entry through TRPV5/6-like channels and subsequently activates CaMKII, which in turn fosters G_1_/S transition, S progression and accumulation in G_2_ phase of the cell cycle (Heise et al., 2010). Moreover, Ca^2+^ signaling in human leukemia cells has been demonstrated to be tightly regulated by voltage-gated K_*v*_3.4 K^+^ channels and translates into G_2_/M cell cycle arrest by CaMKII-mediated inhibitory phosphorylation of the phosphatase cdc25B resulting in inactivation of the mitosis promoting factor and G_2_/M arrest. Radiation activates Kv3.4 currents without changing the surface expression of the channel protein. Most importantly, inhibition of K_*v*_3.4 by tetraethylammonium and blood-depressing substance-1 and substance-2 or silencing of the K_*v*_3.4 channels by RNA interference prevents TRPV5/6-mediated Ca^2+^ entry, CaMKII activation, as well as cdc25B inactivation which results in release from radiation-induced G_2_/M arrest, increased apoptosis, and decreased clonogenic survival. Thus, targeting of K_*v*_3.4 radiosensitizes the leukemia cells demonstrating the pivotal role of this channel in cell cycle arrest required for DNA repair (Palme et al., 2013). Similar results have been obtained in prostate cancer cells, where TRPV6 inhibition by capsaicin resulted in radiosensitization (Klotz et al., 2011).

#### Glucose fueling and chromatin relaxation

In addition to cell cycle control, radiation-induced ion transports are proposed to improve glucose fueling of irradiated tumor cells. Fast proliferating tumor cells have a high metabolism at low external glucose and oxygen concentration in the usually chronically under-perfused growing tumor tissue. At the same time, many tumor cells cover their high energy requirements by anaerobic glycolysis with low ATP yield per metabolized glucose even under normoxic conditions. To sustain sufficient glucose fueling, tumor cells may up-regulate the Na^+^/glucose co-transporter (SGLT). SGLTs are capable to take up glucose into the tumor cell even against a high chemical gradient (Ganapathy et al., 2009). Several tumor entities such as colorectal, pancreatic, lung, head and neck, prostate, kidney, cervical, mammary, and bladder cancer as well as chondrosarcomas and leukemia have indeed been shown to up-regulate SGLTs (Nelson and Falk, 1993; Ishikawa et al., 2001; Helmke et al., 2004; Casneuf et al., 2008; Weihua et al., 2008; Yu et al., 2008; Leiprecht et al., 2011; Wright et al., 2011). The inwardly directed Na^+^ gradient and the voltage across the plasma membrane drive the electrogenic SGLT-generated glucose transport into the cell. The membrane voltage is tightly regulated by the activity of voltage gated K^+^ channels which counteract SGLT-mediated depolarization.

Ionizing radiation has been demonstrated to activate EGF receptors (Dittmann et al., 2009). In addition, SGLT1 reportedly is in complex with and under the direct control of the EGF receptor (Weihua et al., 2008) suggesting radiation-induced SGLT1 modifications. As a matter of fact, ionizing radiation stimulates a long lasting EGFR-dependent and SGLT-mediated glucose uptake in A549 lung adenocarcinoma and head and neck squamous carcinoma cell lines (but not in non-transformed fibroblasts) as shown by ^3^H-glucose uptake and patch-clamp, current clamp recordings (Huber et al., 2012). In the latter experiments, radiation-induced and SGLT-mediated depolarization of membrane potential was preceded by a transient hyperpolarization of the plasma membrane indicative of radiation-induced K^+^ channel activation (Huber et al., 2012). Such radiation-induced increase in K^+^ channel activity has been reported for several tumor cell lines including A549 lung adenocarcinoma cells (Kuo et al., 1993). In this cell line, radiation at doses between 0.1 and 6 Gy stimulates the activity of voltage gated K^+^ channels within 5 min, which gradually declines thereafter. It is tempting to speculate that this radiation-stimulated K^+^ channel activity counteracts the depolarization of the membrane potential caused by the SGLT activity shortly after radiation and sustains the driving force for Na^+^-coupled glucose uptake (Huber et al., 2012).

Ionizing radiation may lead to necrotic as well as apoptotic cell death depending on cell type, dose, and fractionation (Verheij, 2008). In particular, necrotic cell death may be associated with ATP depletion (Dorn, 2013). Increased SGLT activity in irradiated tumor cells might contribute to ATP replenishment counteracting necrotic cell death. Such function has been suggested in irradiated A549 cells by experiments analyzing cellular ATP concentrations, chromatin remodeling, residual DNA damage, and clonogenic survival of irradiated tumor cells (Dittmann et al., 2013). The data demonstrate that radiation of A549 lung adenocarcinoma cells leads to a transient intracellular ATP depletion and to histone H3 modifications crucial for both chromatin remodeling and DNA repair in response to irradiation.

Importantly, recovery from radiation-induced ATP crisis was EGFR/SGLT-dependent and associated with improved DNA-repair and increased clonogenic cell survival. The blockade of either EGFR or SGLT inhibited ATP level recovery and histone H3 modifications. *Vice versa*, inhibition of the acetyltransferase TIP60, which is essential for histone H3 modification, prevented chromatin remodeling as well as ATP crisis (Dittmann et al., 2013). Together, these data suggest that radiation-associated interactions between SGLT1 and EGFR result in increased glucose uptake, which counteracts the ATP crisis in tumor cells caused by chromatin remodeling. Importantly, the blockade of recovery from ATP crisis by SGLT1 inhibition may radio-sensitize tumor cells as demonstrated in lung adenocarcinoma and head and neck squamous carcinoma cell lines (Huber et al., 2012; Dittmann et al., 2013).

#### Formation of repair complexes

In addition to SGLT-generated glucose uptake, radiation-induced electrosignaling via transient receptor potential melastatin 2 (TRPM2) and vanilloid 1 (TRPV1) cation channels, has been shown to stimulate Ataxia telangiectasia mutated (ATM) kinase activation, histone 2AX (H2AX) phosphorylation, and γH2AX focus formation in A549 lung adenocarcinoma cells, processes required to recruit further repair proteins to the DNA double strand break (Masumoto et al., 2013). Furthermore, radiation-induced TRPM2 induces ATP release and P2Y signaling in A549 cells (Masumoto et al., 2013). Radiation-stimulated and P2X_7_ receptor- and gap junction hemichannel connexin43-mediated ATP release has been suggested to signal in a paracrine manner to unirradiated bystander cells in the B16 melanoma model (Ohshima et al., 2012).

Combined, these recent data indicate that ion transports may regulate processes that mediate intrinsic radioresistance. The investigation of ion transports in radiobiology is at its very beginning and the few data available are mostly phenomenological in nature. The molecular mechanisms that underlie, e.g., regulation of DNA repair by ion transports are ill-defined. Nevertheless, the data prove functional significance of ion transports and electrosignaling for the survival of irradiated tumor cells and might have translational implications for radiotherapy in the future.

Similar to intrinsic radioresistance, the function of ion transports in hypoxia resistance and associated hypoxic radioresistance of tumor cells is not well-defined. The following paragraphs give a summary of what is known about mitochondrial transports and hypoxia resistance of normal tissue and how these findings might also apply for tumor cells.

### Mitochondrial uncoupling and resistance to hypoxia, chemo-, and radiotherapy

Intermittent hypoxia is a common feature of vascularized solid tumors. The pathophysiologial aspects of hypoxia and reoxygenation are well-known from ischemia-reperfusion injuries observed in normal tissue. Reoxygenation-associated production of reactive oxygen species (ROS) is a major cause of the hypoxia/reoxygenation injury after myocardial, hepatic, intestinal, cerebral, renal and other ischemia and mitochondria have been identified as one of the main sources of ROS formation herein (Li and Jackson, 2002). Mitochondrial ROS formation mutually interacts with hypoxia/reoxygenation-associated cellular Ca^2+^ overload. Brief hypoxic periods induce an adaptation to hypoxia in several tissues which lowers ischemia-reperfusion injuries of subsequent ischemic insults (so-called ischemic pre-conditioning). Similar adaptations which involve alterations in mitochondrial ion transport have been proposed to confer hypoxia resistance of tumor cells.

#### Mitochondrial ROS formation

**Figure 3:**
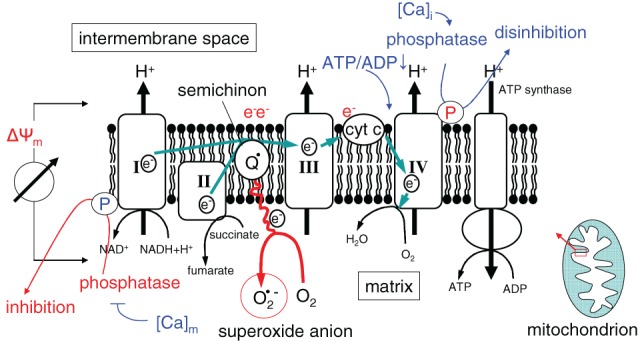
**Mitochondrial ROS formation in dependence on ΔΨ_m_ (I, II, III, and IV, electron transport complexes I, II, III, and IV; Q: semiquinone radical; cyt C: cytochrome C)**. Ca^2+^ and the ATP/ADP ratio regulate the electron chain at complexes I and IV.

Activity and efficacy of the mitochondrial respiration chain are fine-tuned by the dependence of the ATP synthase (complex V) on the membrane potential ΔΨ_m_, by the ATP/ADP ratio, as well as by reversible phosphorylation of the complexes I and IV (Figure 3) (Kadenbach, 2003). It is suggested that under physiological conditions (high ATP/ADP ratios), the membrane potential ΔΨ_m_ is kept low [around −100 to −150 mV (Kadenbach, 2003)]. The efficacy of the respiratory chain at low ΔΨ_m_ is high. At higher ATP demand or decreasing cellular ATP levels, cytochrome c oxidase (complex IV) is relieved from ATP blockade and ΔΨ_m_ increases. High ΔΨ_m_ values (up to −180 mV), however, lower the efficacy of cytochrome c oxidase (Kadenbach, 2003) and increase the probability of single electron leakage at complex I and III to molecular oxygen resulting in an increased O_2_·^−^ production (Figure 3) (Korshunov et al., 1997; Skulachev, 1998; Kadenbach, 2003).

The respiratory chain is also regulated by the cytosolic ([Ca^2+^]_i_) and mitochondrial matrix free Ca^2+^ concentration ([Ca^2+^]_m_) in a complex manner (for review see Pizzo et al., 2012). The phosphatases that dephosphorylate (and thereby switch-off) the NADH oxidase and that relieve the ATP blockade of complex IV are inhibited by [Ca^2+^]_m_ and activated by [Ca^2+^]_i_, respectively (Figure 3). As a consequence, increase in [Ca^2+^]_m_ and [Ca^2+^]_i_ results in a higher ΔΨ_m_ and a concurrently increased production of reactive oxygen species (Kadenbach, 2003).

Hypoxia decreases the activity of the mitochondrial manganese superoxide dismutase (Mn-SOD) and of the cytochrome c oxidase. Depletion of the final electron acceptor, however, increases the formation of O_2_·^−^ during reoxygenation by the enhanced leakage of single electrons from more proximal complexes of the respiration chain (for review see Li and Jackson, 2002; Sack, 2006). Lowered O_2_·^−^-detoxifying capability combined with simultaneous elevated O_2_·^−^ production results in a highly elevated O_2_·^−^ concentration which, e.g., in hepatocytes increases 15-fold within 15 min of reoxygenation (Caraceni et al., 1995).

#### Hypoxia/reoxygenation-associated Ca^2+^ overload

Hypoxia-associated energy depletion and the concomitant impairment of plasma membrane Na^+^ and Ca^2+^ pump activity lead to a decline of the chemical Na^+^, Ca^2+^ and K^+^ gradients across the plasma membrane and to the depolarization of the plasma membrane potential. In parallel, increased lactic acid fermentation during hypoxia increases the cytosolic proton concentration and lowers the intracellular pH. The proton extrusion machinery that is already active during hypoxia becomes massively activated during reoxygenation and restores a physiological pH by wash-out of lactic acid and activation of the sodium/hydrogen exchanger and sodium/bicarbonate symporter. The latter, Na^+^-coupled transports, in turn, further increase the cytosolic Na^+^ concentration to a level, where the low affinity high capacity sodium/calcium exchanger in the plasma membrane starts to operate in the reverse mode (i.e., to extrude Na^+^ at the expense of Ca^2+^ uptake). At that time, reoxygenation-mediated oxidative stress (see above) stimulates further Ca^2+^ entry through Ca^2+^-permeable channels in the plasma membrane and the release of Ca^2+^ from the endoplasmic reticulum resulting in an abrupt rise in [Ca^2+^]_i_ during the first minutes of reoxygenation. Cytosolic Ca^2+^ is buffered by ΔΨ_m_-driven and uniporter-mediated Ca^2+^ uptake into the mitochondrial matrix which increases [Ca^2+^]_m_. Elevated [Ca^2+^]_m_ and [Ca^2+^]_i_ values, in turn, signal back to the respiratory chain by further increasing ΔΨ_m_ (see above). Exceeding the Ca^2+^ threshold concentration in the matrix, [Ca^2+^]_m_ activates the permeability transition pore which leads to breakdown of ΔΨ_m_, swelling of the mitochondrial matrix and eventually release of cytochrome c from the intermembrane space into the cytosol (Crompton, 1999; Rasola and Bernardi, 2011). By reversing the ATP synthase activity, into the ATPase proton pump mode, the F_0_/F_1_ complex in the inner mitochondrial delays the break-down of ΔΨ_m_ at the expense of ATP hydrolysis. In addition to this ATP depletion, the loss of cytochrome c and the concurrent decline of the final electron acceptor (cytochrome c oxidase of complex IV) further increases the formation of O_2_·^−^ by more proximal complexes. The pivotal role of membrane transports in this process is illustrated by the fact that inhibition of the sodium/hydrogen antiporter in the plasma membrane, the Ca^2+^ uniporter in the inner mitochondrial membrane, or Ca^2+^ channels in the endoplasmic reticulum (ER) decreases the hypoxia/reoxygenation injury *in vitro* (for review see Crompton, 1999; Li and Jackson, 2002; Sack, 2006; Yellon and Hausenloy, 2007).

#### Ischemic pre-conditioning

Cells can also adapt to repetitive periods of hypoxia. This so-called ischemic preconditioning has been demonstrated in the myocardium where it reduces ischemia-caused infarct size, myocardial stunning, and incidence of cardiac arrhythmias (Gross and Peart, 2003). Since mitochondrial ROS formation increases with increasing ΔΨ_m_ (Korshunov et al., 1997; Skulachev, 1998; Kadenbach, 2003) lowering of the mitochondrial ΔΨ_m_ is proposed to be a key adaptation event in ischemic preconditioning (Sack, 2006). Lowering of ΔΨ_m_ reduces not only mitochondrial O_2_·^−^ production but also the mitochondrial Ca^2+^ overload during reoxygenation (Gross and Peart, 2003; Prasad et al., 2009). The hypoxic preconditioning-associated reduction of ΔΨ_m_ is in part achieved by up-regulation of ATP-sensitive (mitoKATP) and Ca^2+^-activated (mitoKCa) K^+^ channels in the inner mitochondrial membrane which short-circuit ΔΨ_m_ (Murata et al., 2001; Gross and Peart, 2003; Prasad et al., 2009; Singh et al., 2012; Szabo et al., 2012). The uncoupling proteins-2 and -3 (UCP-2, -3) constitute two further proteins that have been suggested to play a role in counteracting cardiac hypoxia/reoxygenation injury and in hypoxic preconditioning in heart and brain (Mcleod et al., 2005; Sack, 2006; Ozcan et al., 2013). Activation of these proteins results in a modest depolarization of ΔΨ_m_ by maximally 15 mV (Fink et al., 2002). High expression of UCP-3 has also been demonstrated in skeletal muscle where it suppresses mitochondrial oxidant emission during fatty acid-supported respiration (Anderson et al., 2007). Accordingly, overexpression of UCP-3 in cultured human muscle cells lowers ΔΨ_m_, raises the ATP/ADP ratio, and favors fatty acid vs. glucose oxidation (Garcia-Martinez et al., 2001). Conversely, knockdown of UCP-3 increased the coupling between electron and proton transfer across the inner mitochondrial membrane and ROS production (Vidal-Puig et al., 2000; Talbot and Brand, 2005). UCP-3 protein is robustly up-regulated in chondrocytes (Watanabe et al., 2008) and skeletal muscle during hypoxia and the absence of UCP-3 exacerbates hypoxia-induced ROS (Lu and Sack, 2008). UCP-3 is not constitutively active. O_2_·^−^ has been demonstrated to stimulate the activity of UCP-3 in skeletal muscle suggesting that UCP-3 is the effector of a feed back loop which restricts overshooting ROS production (Echtay et al., 2002).

#### Mitochondrial uncoupling in tumor cells

Recent studies suggest that UCPs are upregulated in a number of aggressive human cancers. In particular, over-expression of UCP2 has been reported in leukemia as well as in breast, colorectal, ovarian, bladder, esophagus, testicular, kidney, pancreatic, lung, and prostate cancer (Ayyasamy et al., 2011; Su et al., 2012). In human colon cancer, UCP2 mRNA and protein expression reportedly is increased by factor of 3–4 as compared to peritumoral normal epithelium. In addition, UCP2 expression gradually increases during the colon adenoma-carcinoma sequence (Horimoto et al., 2004) and is higher in clinical stages III and IV colon cancer than in stage I and II (Kuai et al., 2010). Similarly, UCP4 expression has been shown to correlate with lymph node metastases in breast cancer (Gonidi et al., 2011) and UCP1 expression in prostate cancer with disease progression from primary to bone metastatic cancers (Zhau et al., 2011). Moreover, postmenopausal breast tumors with low estrogen receptor (ER) alpha to ER beta ratios that associate with higher UCP5 expression and higher oxidative defense have a poor prognosis (Sastre-Serra et al., 2013). Finally, ectopic expression of UCP2 in MCF7 breast cancer cells has been demonstrated to enhance proliferation, migration and matrigel invasion *in vitro* and to promote tumor growth *in viv*o (Ayyasamy et al., 2011). Together, these observations suggest that UCPs may contribute to the malignant progression of tumor cells.

In addition to malignant progression, UCPs may alter the therapy sensitivity of tumor cells. In specimens of human ovarian cancers carboplatin/paclitaxel-resistant cancers showed decreased UCP2 protein abundances as compared to the sensitive ones (Pons et al., 2012). Likewise, progression-free and overall survival of patients with inoperable lung cancer who received cisplatin-based chemotherapy was higher when tumors expressed high levels of UCP2 as compared to tumors with low UCP2 levels (Su et al., 2012). A possible explanation of the latter observation is that especially in lung tumors with mutated p53, cisplatin elicits oxidative stress that induces pro-survival signaling. High UCP2 expression, however, diminishes cisplatin-evoked oxidative stress and, in turn, decreases the pro-survival signals (Su et al., 2012).

In lung cancer cell lines with wildtype p53, in contrast, downregulation of UCP2 results in significantly increased paclitaxel-induced cell death (Su et al., 2012). Similarly, overexpression of UCP2 in a human colon cancer cell line has been shown to blunt topoisomerase I inhibitor CPT-11-induced accumulation of reactive oxygen species and apoptosis *in vitro* and to confer CPT-11 resistance of tumor *xeno*grafts (Derdak et al., 2008). In addition, in pancreatic adenocarcinoma, non-small cell lung adenocarcinoma, and bladder carcinoma cell lines IC_50_ values of the anticancer drug gemcitabine increase with intrinsic UCP2 mRNA abundance. Furthermore, UCP2 overexpression strongly decreases gemcitabine-induced mitochondrial superoxide formation and protects cancer cells from apoptosis (Dalla Pozza et al., 2012). Finally, metabolic changes including UCP2 up-regulation and UCP2-mediated uncoupling of oxidative phosphorylation have been demonstrated in multidrug-resistant subclones of various tumor cell lines (Harper et al., 2002). Similarly, in acute myeloid leukemia cells, UCP2 up-regulation has been shown to foster the Warburg effect (i.e., anaerobic glycolysis in the absence of respiratory impairment) (Samudio et al., 2008).

UCP2 expression is stimulated by co-culturing of these leukemia cells with bone marrow-derived mesenchymal stromal cells (Samudio et al., 2008). Other stimuli of UCP expression/activity are hydrogen peroxide as shown for UCP5 in colon cancer cells (Santandreu et al., 2009) and gemcitabine chemotherapy as reported for UCP2 in pancreatic, lung and bladder cancer cell lines (Dalla Pozza et al., 2012). Collectively, these data suggest that tumor cells may acquire resistance to chemotherapy by up-regulation of UCPs and lowering of the therapy-evoked mitochondrial formation of reactive oxygen species (Robbins and Zhao, 2011).

Accordingly, experimental targeting of UCPs has been demonstrated to sensitize tumor cells to chemotherapy *in vitro*. For instance, genipin-induced inhibition or glutathionylation of UCP2 sensitizes drug-resistant leukemia subclones to chemotherapy with menadione, doxorubicin, or epirubicin (Mailloux et al., 2010; Pfefferle et al., 2012). Likewise, UCP2 inhibition by genipin or UCP2 mRNA silencing strongly enhances gemcitabine-induced mitochondrial superoxide generation and apoptotic cell death of pancreatic, lung and bladder cancer cell lines (Dalla Pozza et al., 2012). Moreover, UCP2 inhibition has been reported to trigger reactive oxygen species-dependent nuclear translocation of GAPDH and autophagic cell death in pancreatic adenocarcinoma cells (Dando et al., 2013) Together, this suggests that targeting UCPs might be a promising strategy to overcome resistance to anti-cancer therapies in the clinic. Notably, in an acute myeloid leukemia cell line, the cytotoxicity of cisplatin has been proposed to be in part mediated by cisplatin-dependent down-regulation of UCPs (Samudio et al., 2008) suggesting that established chemotherapy regimes already may co-target UCPs.

It is tempting to speculate that UCPs may also confer resistance to radiotherapy. One could hypothesize that UCPs adapt the tumor cells to a “relatively radioprotected” hypoxic microenvironment by decreasing hypoxia-associated mitochondrial formation of reactive oxygen species. Such UCP function in hypoxia resistance has been demonstrated for a lung adenocarcinoma cell line (Deng et al., 2012). Notably, radiation induces up-regulation of UCP2 expression as shown in colon carcinoma cells (Sreekumar et al., 2001) and in a radiosensitive subclone of B cell lymphoma (Voehringer et al., 2000). On the one hand, this UCP2 up-regulation might facilitate radiation-induced apoptosis induction by accelerating the break-down of ΔΨ_m_ as proposed by the authors of these studies. On the other hand, radiation-induced UCP2 upregulation might be radioprotective by lowering the radiation-induced burden of reactive oxygen species. As a matter of fact, multi-resistant subclones of leukemia cells show higher UCP2 protein expression, lower ΔΨ_m_, lower radiation induced formation of reactive oxygen species and decreased DNA damage as compared to their parental sensitive cells (Harper et al., 2002).

In summary, UCPs suppress the formation of O_2_·^−^, a byproduct of the mitochondrial respiration chain and a major source of oxidative stress. In some cancers UCPs in particular UCP2 are highly upregulated and may contribute to the reprogramming of the cell metabolism that results in chemoresistance (for review see Baffy, 2010; Baffy et al., 2011) or even radioresistance. Moreover, recent studies imply that UCP2 may repress p53-mediated apoptosis providing a potential new mechanism of how UCP2 contributes to cancer development (Robbins and Zhao, 2011).

Together, these observations suggest that ion transport processes are critically involved in evasion from radiation stress, and intrinsic or hypoxic radioresistance. Since ion transport-mediated radioresistance might underlie failure of radiotherapy, concepts which combine ion transport targeting with radiotherapy hold promise for new therapy strategies in the future. A summary of how ion transport can be harnessed for anticancer therapy and how these therapy strategies might be combined with radiotherapy is given in the next paragraphs.

## Targeting ion transports in radiotherapy

An important reason for the study of ion transports in the context of radiotherapy is the possible translation of the acquired knowledge into anti-cancer therapy. Many pharmacological modulators of ion transports are already in clinical use or currently tested in clinical trials (Wulff and Castle, 2010). Moreover, tumors often over-express certain types of transport proteins.

These proteins such as the transient receptor melastatin 8 (TRPM8) non-selective cation channel in prostate cancer have been used in clinical trials as tumor-associated antigen for anti-tumor vaccination (Fuessel et al., 2006). Tumor promoting inflammation and anti-tumor immune effects are evolving fields of preclinical and clinical research (Hanahan and Weinberg, 2011). Preclinical evidence supports the thesis that tumors have to develop immune-evading capacities in order to grow into macroscopic, clinically detectable lesions (Koebel et al., 2007; Teng et al., 2008). Possible mechanisms are the secretion of cytokines and chemokines by cancer and tumor stroma cells (Vianello et al., 2006; Shields et al., 2010), the priming of infiltrating T-lymphocytes toward immunosuppressive regulatory T-cells and the recruitment of myeloid-derived suppressor cells and tumor-associated macrophages (Tanchot et al., 2013; Oleinika et al., 2013). Irradiation of tumors has been shown to impair on the one hand the immunosuppressive action of the tumor and on the other to induce so-called “immunogenic” cell death within the tumor with translocation of calreticulin to the plasma membrane, release of HMGB1 or ATP (Formenti and Demaria, 2013). Preclinical studies showed a synergistic effect of irradiation and several immunotherapeutic approaches such as dendritic cell injection (Finkelstein et al., 2012), anti-CTLA-4-antibody (Grosso and Jure-Kunkel, 2013), and vaccines (Chakraborty et al., 2004). Interestingly, for combination with anti-CTLA-4 antibody a synergistic effect could only be demonstrated for fractionated but not for single-dose irradiation (Demaria and Formenti, 2012).

In addition, over-expressed transport proteins in tumors can be harnessed to target drugs, cytokines, or radioactivity to the tumor cells (Hartung et al., 2011). One example is the specific surface expression of ClC-3 Cl^−^ channels by glioblastoma (and other tumor entities) which suggests ClC-3 as an excellent and highly specific target for anti-glioblastoma therapy. Chlorotoxin which is a 36 amino acid-long peptide from the venom of the scorpion *Leiurus quinquestriatus* has been found to inhibit ClC-3 and to preferentially bind to the cell surface of a variety of human malignancies. This specificity probably comes from the highly affine binding of chlorotoxin to a lipid raft-anchored complex of matrix metalloproteinase-2, membrane type-I MMP, and transmembrane inhibitor of metalloproteinase-2, as well as ClC-3 (Veiseh et al., 2007). Ongoing clinical trials successfully used ^131^I-labeled chlorotoxin as glioblastoma-specific PET-tracer (Hockaday et al., 2005) and for targeted radiation of glioblastoma cells (Mamelak and Jacoby, 2007). Due to the low surface expression of ClC-3 in normal tissue, chlorotoxin exhibits little or no affinity to normal cells (Lyons et al., 2002). If the *in vitro* and mouse data on radiation-stimulated glioblastoma migration reflect indeed the *in vivo* situation in glioblastoma patients, a clinical setting might be envisaged in which radiation-induced glioblastoma spreading is prevented by combining radiotherapy with chlorotoxin blockade of ClC-3 channels.

## Concluding remarks

Interdisciplinary approaches linking radiobiology with physiology brought about the first peaces of evidence suggesting a functional significance of ion transport processes for the survival of irradiated tumor cells. The few reports published up to now on this topic are confined to phenomena occurring in the plasma membrane due to the methodological restrictions of studying these processes in the membranes of mitochondria, endoplasmic reticulum, or nuclear envelope. Intracellular membrane transports, however, might similarly impact tumor cell radiosensitivity. This is suggested by the notion that intracellular Cl^−^ channel CLIC1 protein expression regulates radiosensitivity in laryngeal cancer cells (Kim et al., 2010). However, the molecular mechanisms underlying, e.g., radiation-induced transport modifications, or downstream signaling events are far from being understood. Despite all these limitations, our current knowledge already clearly indicates that the observed transport processes may be crucial for the survival of the tumor and, thus, are worthwhile to spend further and more effort in this field which might lead to new strategies for cancer treatment in the future.

### Conflict of interest statement

The authors declare that the research was conducted in the absence of any commercial or financial relationships that could be construed as a potential conflict of interest.
